# Role of thioredoxin reductase 1 and thioredoxin interacting protein in prognosis of breast cancer

**DOI:** 10.1186/bcr2599

**Published:** 2010-06-28

**Authors:** Cristina Cadenas, Dennis Franckenstein, Marcus Schmidt, Mathias Gehrmann, Matthias Hermes, Bettina Geppert, Wiebke Schormann, Lindsey J Maccoux, Markus Schug, Anika Schumann, Christian Wilhelm, Evgenia Freis, Katja Ickstadt, Jörg Rahnenführer, Jörg I Baumbach, Albert Sickmann, Jan G Hengstler

**Affiliations:** 1 Department of Bioanalytics, Leibniz-Institut für Analytische Wissenschaften - ISAS - e.V., Bunsen-Kirchhoff-Straße 11, Dortmund 44139, Germany; 2 Systems Toxicology, Leibniz Research Centre for Working Environment and Human Factors (IfADo) at the TU Dortmund University, Ardeystraße 67, Dortmund 44139, Germany; 3 Department of Obstetrics and Gynecology, University Medical Center, Langenbeckstraße 1, Mainz 55131 Germany; 4 H DX DB Molecular Research Koeln, Siemens Healthcare Diagnostics Products GmbH, Nattermannallee 1, Koeln 50829, Germany; 5 Department of Plant Physiology, University of Leipzig, Johannisallee 21-23, Leipzig 04103, Germany; 6 Department of Statistics, TU Dortmund University, Vogelpothsweg 87, Dortmund 44221, Germany

## Abstract

**Introduction:**

The purpose of this work was to study the prognostic influence in breast cancer of thioredoxin reductase 1 (TXNRD1) and thioredoxin interacting protein (TXNIP), key players in oxidative stress control that are currently evaluated as possible therapeutic targets.

**Methods:**

Analysis of the association of *TXNRD1 *and *TXNIP *RNA expression with the metastasis-free interval (MFI) was performed in 788 patients with node-negative breast cancer, consisting of three individual cohorts (Mainz, Rotterdam and Transbig). Correlation with metagenes and conventional clinical parameters (age, pT stage, grading, hormone and ERBB2 status) was explored. MCF-7 cells with a doxycycline-inducible expression of an oncogenic ERBB2 were used to investigate the influence of ERBB2 on *TXNRD1 *and *TXNIP *transcription.

**Results:**

TXNRD1 was associated with worse MFI in the combined cohort (hazard ratio = 1.955; *P *< 0.001) as well as in all three individual cohorts. In contrast, TXNIP was associated with better prognosis (hazard ratio = 0.642; *P *< 0.001) and similar results were obtained in all three subcohorts. Interestingly, patients with ERBB2-status-positive tumors expressed higher levels of *TXNRD1*. Induction of ERBB2 in MCF-7 cells caused not only an immediate increase in *TXNRD1 *but also a strong decrease in *TXNIP*. A subsequent upregulation of *TXNIP *as cells undergo senescence was accompanied by a strong increase in levels of reactive oxygen species.

**Conclusions:**

*TXNRD1 *and *TXNIP *are associated with prognosis in breast cancer, and ERBB2 seems to be one of the factors shifting balances of both factors of the redox control system in a prognostic unfavorable manner.

## Introduction

Control mechanisms of reactive oxygen species (ROS) play a crucial role in tumor development. Transformed cells are known to generate more ROS than normal cells [1,2]. Importantly, ROS not only contribute to tumor progression by amplifying genomic instability but transformed cells use ROS signals to drive proliferation [1]. Conversely, ROS addiction may render tumor cells more vulnerable to apoptosis or senescence [3,4] because they depend on constantly increased basal levels of ROS, and an additional increase may exceed toxic thresholds.

Thioredoxin reductase 1 (TXNRD1) and thioredoxin interacting protein (TXNIP; also called thioredoxin binding protein 2 or vitamin D3-upregulated protein 1) [5] are key players in oxidative stress control.

TXNRD1 reduces and activates thioredoxin, an oxidoreductase containing a dithiol-disulfide active site, which in turn reduces oxidized cysteine residues on cellular proteins. Importantly, a reducing environment mediated by thioredoxin is required for effective DNA binding of redox-sensitive transcription factors, including p53 and NF-κB [6,7]. Thioredoxin binds ROS before they can harm cells and thus protects cells against oxidative stress. In addition to its critical role in the regulation of cellular redox homeostasis, thioredoxin has multiple actions in the cell - such as activation of ribonucleotide reductase, inhibition of apoptosis signal regulating kinase 1 and induction of hypoxia inducible factor 1 (HIF-1) and vascular endothelial growth factor (VEGF) - which contribute to many hallmarks of cancer, such as increased proliferation, inhibited apoptosis and angiogenesis [8].

**Figure 1:**
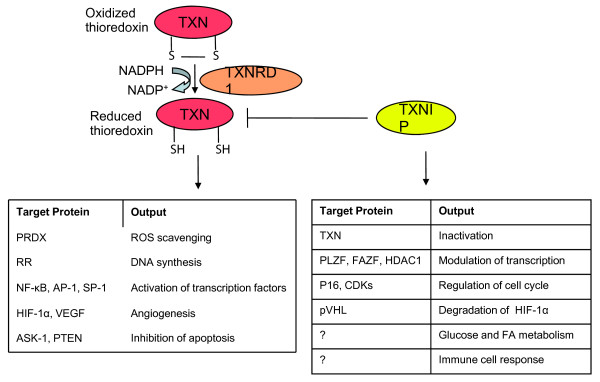
**Scheme of cellular functions of the cytoplasmic thioredoxin system**. Thioredoxin reductase 1 (TXNRD1) reduces thioredoxin 1 (TXN), which in turn reduces oxidized cysteins in cellular proteins and scavenges peroxides by peroxiredoxins (PRDX), thus protecting cells against oxidative stress. TXN stimulates ribonucleotide reductase (RR) activity and supports DNA synthesis. TXN stimulates the transcriptional activity of nuclear factor κB (NF-κB), activator protein 1 (Fos/Jun) (AP-1) and specificity protein 1 (SP-1), and elevates hypoxia inducible factor 1α (HIF-1α) protein levels, which in turn influence vascular endothelial growth factor (VEGF). TXN also binds to and inhibits the pro-apoptotic proteins apoptosis signal regulating kinase 1 (ASK-1) and phosphatase and tensin homolog (PTEN). All these events promote cell growth, inhibit apoptosis and increase angiogenesis in tumors, as reviewed elsewhere [8]. Thioredoxin interacting protein (TXNIP) binds and inhibits reduced TXN. TXNIP also interacts with several transcriptional co-repressors, such as promyelocytic leukemia zinc-finger (PLZF), Fanconi anemia zinc-finger (FAZF) and histone deacetylase 1 (HDAC1), and inhibits transcription of cyclin A_2_, leading to reduced cell growth. TXNIP increases p16 and p27KIP1 protein levels, leading to cell cycle arrest. TXNIP also associates with the von Hippel-Landau protein (pVHL), enhancing the interaction between pVHL and HIF1α to promote nuclear export and degradation of HIF1α. The roles of TXNIP in the immune system and in human metabolism are not yet fully understood. For reviews, see [13,14]. CDK, cycline dependent kinase; FA, fatty acid; ROS, reactive oxygen species.

In contrast to TXNRD1, which supports thioredoxin function, TXNIP binds to and inhibits the reduced form of thioredoxin [9-11], blocking its activity as well as its interaction with other factors, including apoptosis signal regulating kinase 1. TXNIP therefore functions as a proapoptotic protein [12]. As for thioredoxin, multifunctional roles of TXNIP are known [13,14] that point out the crucial role of TXNIP as a link between pathways of redox regulation, antioxidant defense, energy metabolism and cell growth and survival [15,16]. A summary of the roles of these key players of the thioredoxin system is provided in Figure 1.

The thioredoxin redox system has been suggested recently as a therapeutic target for cancer therapy [17,18], based on the observation that thioredoxin is overexpressed in many aggressive tumors and that siRNA-mediated knockdown of TXNRD1 decreased tumor progression and metastasis in mice [19]. Several inhibitors of the thioredoxin pathway have been shown to have antitumor activity in mice bearing breast, colon and renal xenografts [20-22]. In addition, high thioredoxin expression in prechemotherapy tumor samples has been reported to be associated with resistance to docetaxel in primary breast cancer [23,24].

Despite the ongoing evaluation of the thioredoxin system as a therapeutic target and the central role of TXNRD1 and TXNIP in oxidative stress control, little is known about their prognostic relevance. Increased expression of thioredoxin in human colorectal cancer is associated with decreased patient survival [25], whereas absence of thioredoxin expression in nonsmall-cell lung carcinoma is associated with a better outcome [26]. Decreased TXNIP expression in patients with diffuse large B-cell lymphoma has been shown to correlate with a poor prognosis [27]. To our knowledge, however, TXNRD1 expression and TXNIP expression have not yet been analyzed in relation to prognosis in breast cancer. In the present study we observed that high expression of TXNRD1 and low expression of TXNIP are associated with worse prognosis in breast cancer. Since both higher TXNRD1 expression and lower TXNIP expression were observed in ERBB2-status-positive tumors, we analyzed whether ERBB2 can influence these factors using a MCF-7 cell line that allows conditional expression of an oncogenic version of ERBB2.

## Materials and methods

### Cultivation of MCF-7/NeuT cells and analysis of RNA expression patterns

The MCF-7 breast carcinoma cell line was obtained from the American Type Culture Collection (LGC Standards GmbH, Wesel, Germany), cultured at 37°C in a humidified 5% CO_2 _air atmosphere, and transfected with pINSpBI-NeuT/EGFP and pcDNA3Neo/rtTA2 as described elsewhere [3]. Expression of NeuT, an oncogenic version of ERBB2, was induced by doxycycline (obtained as the hydrochloride salt from Sigma, Munich, Germany) at a final concentration of 1 μg/ml in all experiments. MCF-7/NeuT cells were exposed to doxycycline for periods of 0, 6, 12 and 24 hours as well as 3 and 14 days. Three independent repeat experiments were performed. Between each repeat experiment, the MCF-7/NeuT cells were cultivated for at least two passages. Cells were harvested using TRItidy G-Reagent (AppliChem GmbH, Darmstadt, Germany) and RNA was isolated subsequently according to the manufacturer's protocols and stored at -80°C.

Before microarray analysis, RNA integrity and concentration were examined using an Agilent 2100 Bioanalyzer (Agilent Technologies, Palo Alto, CA, USA) with the RNA 6000 LabChip Kit (Agilent Technologies) according to the manufacturer's instructions. Microarray analysis was conducted at the microarray core facility of the Interdisziplinäres Zentrum für klinische Forschung (Faculty of Medicine, University of Leipzig, Germany). To create cDNA, the High Capacity cDNA Reverse Transcription Kit was used (Applied Biosystems, Darmstadt, Germany). The TaqMan technique was used for further gene expression analysis by quantitative real-time PCR and confirmation of microarray data. Ubiquitin C was chosen as the reference gene and the untreated cells were taken as the controls. The expression assays used were Hs00824723_m1 (*ubiquitin C*), Hs00197750_m1 (*TXNIP*) and Hs00917067_m1 (*TXNRD1*) (Applied Biosystems). The PCR conditions followed the standard specifications recommended by Applied Biosystems. For calculations of relative gene expression, we used the 2^-ΔΔ*Ct *^method as described by Schug and colleagues [28].

### Immunoblotting

Immunoblot analysis was performed as described elsewhere [3,29]. The Neu antibody (sc-284; Santa Cruz Biotechnology, Heidelberg, Germany) and the p44/42 mitogen-activated protein kinase antibody (#9102; Cell Signaling, Boston, MA, USA) were diluted at 1:1,000 and were incubated for 2 hours at room temperature. The phospho-p44/p42 mitogen-activated protein kinase Tyr202/Thr204 antibody (#9101; Cell Signaling) and the phospho-Akt Ser473 (#9271; Cell Signaling) were diluted at 1:1,000 and incubated overnight at 4°C. The phospho-p38 mitogen-activated protein kinase Thr180/Tyr182 (3D7) (#9215; Cell Signaling) was diluted at 1:500 and incubated overnight at 4°C. The Txnip (Vdup1) (sc-166234; Santa Cruz) antibody was diluted 1:250 and the Txnrd1 antibody (ab 16840; Abcam, Cambridge, UK) was diluted 1:1,000. Both antibodies were applied overnight at 4°C. The β-actin antibody (clone AC-74; Sigma Aldrich, Munich, Germany) was diluted 1:5,000 and was incubated for 30 minutes at room temperature. Horseradish peroxidase-conjugated secondary antibodies were obtained from Cell Signaling (anti-rabbit-HRP, #7074) or from Sigma (anti-mouse-HRP, A9044) and were diluted 1:10,000 in 5% bovine serum albumin/Tris-bufffered saline Tween 20. Protein signals were detected by enhanced chemiluminiscence (PerkinElmer LAS, Rodgau-Jügesheim, Germany).

### Detection of malondialdehyde as a thiobarbituric acid or an *N*-methyl-2-phenylindole complex

Malondialdehyde (MDA) is a major degradation product of lipid hydroperoxides and is widely used as a marker of lipid peroxidation. To estimate the content of MDA in a sample we performed two analytical methods.

First, we measured thiobarbituric acid reactive substances via high-performance liquid chromatography according to the procedure described [30]. Aqueous trichloroacetic acid in the presence of hexane and butylated hydroxytoluene was used to homogenize the sample. Within this reaction a complex of MDA and 1,3-diethyl-2-thiobarbituric acid (DETBA) is formed. The chromophore of the MDA-DETBA adduct was then chromatographed on a high-performance liquid chromatography column and quantified by fluorescence emission at 553 nm with an excitation at 532 nm.

Second, we used a spectrophotometric assay based on a reaction of MDA with *N*-methyl-2-phenylindole (Bioxytech MDA-586; Oxis Research, Foster City, CA, USA). One molecule of MDA reacts with two molecules of *N*-methyl-2-phenylindole. The resulting stable carbocyanine dye shows a maximum absorption at 586 nm and was detected spectrophotometrically. For MDA estimation, eight measurements of three independent experiments were performed.

### Patient characteristics, tissue specimens and gene array analysis

Three recently described cohorts of 788 node-negative breast cancer patients who did not receive chemotherapy were analyzed [31]. The combined cohort consisted of three individual cohorts: the Mainz cohort (*n *= 200), the Rotterdam cohort (*n *= 286) and the Transbig cohort (*n *= 302).

The Mainz study cohort consists of 200 lymph node-negative breast cancer patients treated at the Department of Obstetrics and Gynecology of the Johannes Gutenberg University Mainz between 1988 and 1998 [31]. Patients of the Mainz cohort were all treated with surgery and did not receive any systemic therapy in the adjuvant setting. The established prognostic factors (histological grade, tumor size, age at diagnosis, and steroid receptor status) were collected from the original pathology reports of the gynecologic pathology division within our department. Patients' characteristics have been published by Schmidt and colleagues [31] and are described in Additional file 1. For all tumors, samples were snap-frozen and stored at -80°C. Gene expression profiling of the patients' RNA was performed using the Affymetrix HG-U133A array and the GeneChip System as described [31]. These data have been deposited in the National Center for Biotechnology Information Gene Expression Omnibus (GEO) and are accessible [GEO:GSE11121].

Results obtained from the Mainz cohort were validated in two previously published microarray datasets. Two breast cancer Affymetrix HG-U133A microarray datasets including patient outcome information were downloaded from the National Center for Biotechnology Information GEO data repository. The first dataset, the Rotterdam cohort [32], represents 180 lymph node-negative relapse-free patients [GEO:GSE2034] and 106 lymph node-negative patients that developed a distant metastasis. None of these patients had received systemic neoadjuvant or adjuvant therapy (Rotterdam cohort). The original data were recalculated to a mean target intensity (TGT) of 500. The second dataset, the Transbig cohort, consists of 302 samples from breast cancer patients that remained untreated in the adjuvant setting after surgery [33,34]. GEO sample record numbers of samples [GEO:GSE6532, GEO:GSE7390] used for analysis are listed in the supplementary tables previously published by Schmidt and colleagues [31]. Raw .cel file data were processed by MAS 5.0 using a TGT of 500.

Ethical approval for the analysis of RNA levels was required and granted by the local ethical committee (Landesärztekammer Rheinland-Pfalz, reference 837.139.05(4797)).

### Statistical analysis

Univariate and multivariate Cox models were applied to analyze a possible association of TXNRD1 and TXNIP RNA expression (log_2 _transformed) with prognosis. Disease-free survival was computed from the date of diagnosis to the date of local recurrence of disease, or distant metastasis, or cancer of the contralateral breast, or death from cancer. The metastasis-free survival interval (MFI) was computed from the date of diagnosis to the date of diagnosis of distant metastasis. Patients who died of an unrelated cause were censored at the date of death. Survival times were compared using Kaplan-Meier plots and the log-rank test. Since the frequency distributions of TXNRD1 and TXNIP did not perfectly match a normal distribution (Additional file 2) we used nonparametric tests for comparison of groups, such as the Mann-Whitney test for unpaired data.

Dichotomization was performed as described by Schmidt and colleagues [35,36]. Briefly, the cut-off points differentiating between high and low expression were identified in the combined cohort of patients from Mainz, from Rotterdam and from the Transbig group (Additional file 3). Similar bimodal distributions and cut-off points were observed in each individual cohort (Additional file 4). Cut-off points of 10 (estrogen receptor), 12.6 (ERBB2) and 4.9 (progesterone receptor) could therefore be used for all cohorts. Dichotomization was performed using these cut-off points to generate the following dichotomous variables: ERBB2 status, estrogen receptor status, progesterone receptor status and hormone receptor status (which is positive when only one of both estrogen or progesteron receptor status is positive).

Concerning histological grading, the patients were dichotomized into grade III versus grade I and grade II, and pT stage into pT_2 _and pT_3 _(> 2 cm) versus pT_1 _(≤ 2 cm) (for patient characteristics see Additional file 1).

Correlations were analyzed using the Spearman correlation test. All *P *values are two-sided. As no correction for multiple testing was performed, the values should be regarded as descriptive measures. All analyses were performed using SPSS17.0 (SPSS Inc., Chicago, IL, USA).

## Results

### TXNDR1 and TXNIP are associated with prognosis in breast cancer

To study a possible association with the MFI, we analyzed our cohort (Mainz cohort) of patients and validated the results in two previously published cohorts (Rotterdam and Transbig cohorts). All patients were node-negative and did not receive chemotherapy. In the Mainz cohort, high *TXNRD1 *RNA expression was associated with a higher hazard ratio (HR = 1.920; *P *= 0.032) of metastasis (Table 1). In contrast, *TXNIP *was associated with a decreased HR (HR = 0.598; *P *= 0.017) (Table 1). Similar results were obtained in the Rotterdam cohort, the Transbig cohort and the combined cohorts (Table 1).

**Table 1:** Association of *TXNRD1 *and *TXNIP *RNA expression with metastasis-free survival

	Prognostic factor	*P *value	HR	95% CI
Mainz cohort (*n *= 200)	*TXNRD1 *RNA expression	0.032	1.920	1.058 to 3.486
	*TXNIP *RNA expression	0.017	0.598	0.392 to 0.912
Rotterdam cohort (*n *= 286)	*TXNRD1 *RNA expression	0.004	1.755	1.196 to 2.576
	*TXNIP *RNA expression	< 0.001	0.654	0.525 to 0.815
Transbig cohort (*n *= 302)	*TXNRD1 *RNA expression	0.021	1.692	1.084 to 2.642
	*TXNIP *RNA expression	0.013	0.639	0.448 to 0.910
Combined cohort (*n *= 788)	*TXNRD1 *RNA expression	< 0.001	1.955	1.519 to 2.518
	*TXNIP *RNA expression	< 0.001	0.642	0.554 to 0.743

Thioredoxin reductase 1 (*TXNRD1*) RNA expression is associated with worse metastasis-free survival in patients with node-negative breast cancer in the univariate Cox analysis. In contrast, thioredoxin interacting protein (*TXNIP*) is associated with better metastasis-free survival. Results obtained in the Mainz cohort could be validated in the Rotterdam cohort and in the Transbig cohort of node-negative breast cancer patients. In the combined cohort of 788 patients, significant associations of TXNRD1 and TXNIP with metastasis-free survival were also obtained. HR: hazard ratio, CI, confidence interval.

Besides MFI, which captures the time between surgery and the occurrence of a distant metastasis, disease-free survival and overall survival were documented in the Mainz cohort. Disease-free survival comprises metastasis as well as local recurrence or carcinomas at the contralateral breast, whereas overall survival represents the time interval until breast-cancer-specific death. When analyzing disease-free survival and overall survival (Table 2), significant associations with TXNRD1 and TXNIP were also obtained.

**Table 2:** Association of *TXNRD1 *and *TXNIP *RNA with disease-free survival and overall survival

Survival	Prognostic factor	*P *value	HR	95% CI
Disease-free	*TXNRD1 *RNA expression	0.010	2.025	1.180 to 3.476
	*TXNIP *RNA expression	0.012	0.617	0.423 to 0.899
Overall	*TXNRD1 *RNA expression	0.032	1.838	1.054 to 3.204
	*TXNIP *RNA expression	0.192	0.774	0.526 to 1.137

In patients from the Mainz cohort (*n *= 200) of node-negative breast cancer in the univariate Cox analysis, an association of thioredoxin reductase 1 (*TXNRD1*) and thioredoxin interacting protein (*TXNIP*) RNA with disease-free survival and overall survival was observed. HR, hazard ratio; CI, confidence interval.

**Figure 2:**
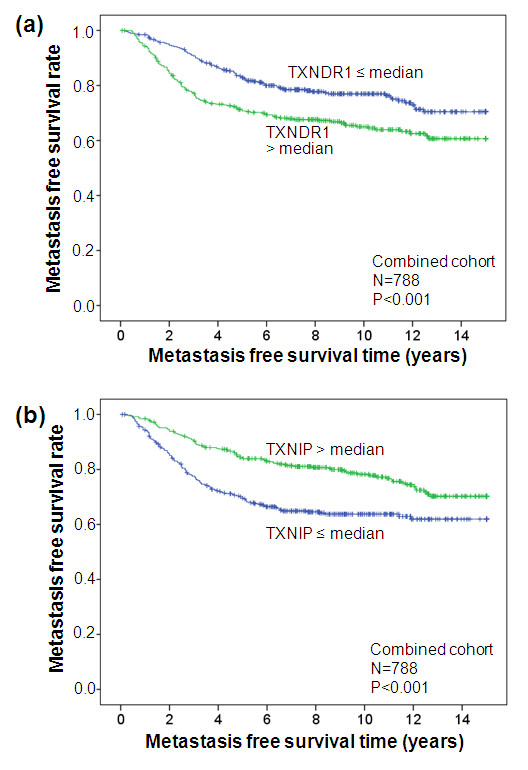
**Association of TXNRD1 and TXNIP with metastasis-free survival time**. **(a) **High thioredoxin reductase 1 (TXNDR1) expression is associated with shorter metastasis-free survival time, **(b) **whereas high thioredoxin interacting protein (TXNIP) expression is associated with longer metastasis-free survival time. The analysis included 788 patients with node-negative breast cancer who have not been treated by chemotherapy. Both TXNDR1 and TXNIP were dichotomized at the median. The log-rank test was used to assess statistical significance of the Kaplan-Meier plots.

Kaplan-Meier analysis was used to visualize the association between metastasis-free survival time and *TXNDR1 *as well as *TXNIP *(Figure 2). The time interval until occurrence of metastasis was shorter for patients with *TXNRD1 *expression higher than the median compared with patients with relatively low expression levels (Figure 2a). In contrast, patients with high *TXNIP *had longer MFI compared with patients with low *TXNIP *(Figure 2b).

In conclusion, the influence of TXNRD1 and TXNIP could be confirmed in three independent cohorts of node-negative breast cancer patients.

### TXNDR1 and TXNIP in relation to known biological motives in breast cancer

**Figure 3:**
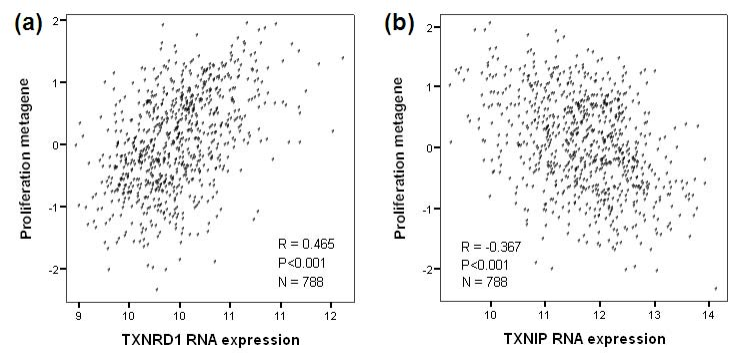
**Correlation of TXNRD1 and TXNIP with the proliferation metagene**. **(a) **Thioredoxin reductase 1 (TXNDR1) shows a positive correlation with the proliferation metagene, **(b) **whereas thioredoxin interacting protein (TXNIP) inversely correlates with the proliferation metagene. The analysis includes carcinomas of 788 patients with node-negative breast cancer.

In recent years, the genome-wide search for markers predicting prognosis in breast cancer has led to a global picture in which three coordinates representing important biological processes have outstanding prognostic consequences [31,35,36]: the proliferation metagene, consisting of a group of genes indicating transition from slow to fast proliferation; the B-cell and T-cell metagenes as markers for immune cell infiltration; and estrogen-receptor-dependent genes. In order to understand the role of TXNRD1 and TXNIP, we determined their relation to these three coordinates according to Freis and colleagues [37]. *TXNRD1 *showed a positive correlation (*R *= 0.465; *P *< 0.001) (Figure 3a) and *TXNIP *an inverse correlation (*R *= -0.367; *P *< 0.001) with the proliferation metagene (Figure 3b). In addition, *TXNRD1 *showed an inverse correlation with the estrogen receptor metagene (*R *= -0.432; *P *< 0.001) and a weakly positive correlation with the T-cell metagene (*R *= 0.230; *P *< 0.001). TXNIP showed also a weakly positive correlation with the B-cell metagene (*R *= 0.278; *P *< 0.001).

### TXNRD1 is associated with ERBB2 status

To understand whether TXNRD1 and TXNIP are independent of conventional clinical parameters, we performed a multivariate Cox analysis (Table 3). For this purpose the regression model is usually adjusted for age, pT stage, grading, hormone (estrogen and progesterone) receptor as well as ERBB2 status [38]. In multivariate analysis, only *TXNIP *was independent of the clinical parameters (*P *= 0.037; HR = 0.654) whereas *TXNRD1 *was not (Table 3). The reason for a lack of significance of *TXNRD1 *in the multivariate regression model was its association with higher pT stage (*P *< 0.001), higher grading (*P *< 0.001), negative hormone receptor status (*P *< 0.001) and positive ERBB2 status (*P *< 0.001) (Additional file 5).

**Table 3:** Multivariate analysis of the association between *TXNRD1 *and *TXNIP *RNA expression with metastasis-free survival

Prognostic factor	*P *value	HR	95% CI
Age (< 50 years vs. ≥50 years)	0.511	1.176	0.725 to 1.910
pT stage (≤2 cm vs. >2 cm)	< 0.001	3.526	1.866 to 6.661
Histological grade (grade III vs. grades I and II)	0.223	0.708	0.406 to 1.234
Hormone receptor^a^, ER or PR (negative vs. positive)	0.865	0.951	0.529 to 1.707
ERBB2 status (positive vs. negative)	0.449	1.276	0.679 to 2.399
*TXNRD1 *RNA expression	0.162	1.445	0.862 to 2.420
*TXNIP *RNA expression	0.037	0.654	0.439 to 0.974

The analysis was carried out in patients with node-negative breast cancer in the univariate Cox analysis (Transbig cohort, n = 302). HR, hazard ratio; CI, confidence interval; *TXNRD1*, thioredoxin reductase 1; *TXNIP*, thioredoxin interacting protein. ^a^The hormone receptor status (HR) is positive as soon as either the estrogen receptor (ER) status or the progesterone receptor (PR) status is positive.

**Figure 4:**
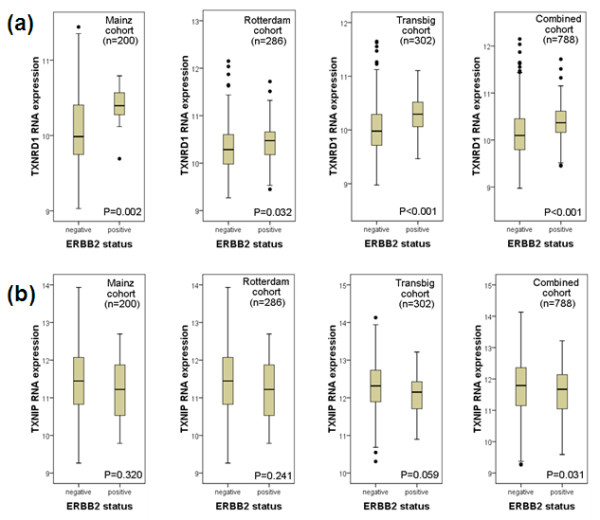
**Association of ERBB2 status with TXNRD1 and TXNIP expression**. **(a) **Positive ERBB2 status is associated with significantly higher levels of thioredoxin reductase 1 (TXNDR1) expression. A significant association was obtained in all three individual cohorts and in the combined cohort including 788 patients with node-negative breast cancer. **(b) **In contrast to TXNDR1, a trend towards lower thioredoxin interacting protein (TXNIP) expression was obtained in ERBB2-status-positive carcinomas. This trend, however, amounted to statistical significance only in the combined cohort.

Because of the high clinical relevance of ERBB2, its correlation with TXNRD1 seemed particularly interesting and was analyzed in detail (Figure 4a). Higher *TXNRD1 *expression in ERBB2-status-positive patients compared with ERBB2-status-negative patients was observed in the Mainz cohort. This result was confirmed in the Rotterdam cohort and in the Transbig cohort (Figure 4a). In contrast to TXNRD1, the association with ERBB2 status was much weaker for *TXNIP *(Figure 4b). A trend towards lower *TXNIP *expression in ERBB2-status-positive patients was obtained that amounted to statistical significance only in the combined cohort with 788 patients (Figure 4b).

In conclusion, *TXNRD1 *showed a positive association and *TXNIP *a trend towards an inverse association with ERBB2 status.

### Other members of the thioredoxin pathway are also associated with prognosis in breast cancer

The findings above prompted us to investigate whether other members of the thioredoxin pathway and downstream factors are also associated with prognosis of breast cancer. For this purpose we focused on the most prominent members of the thioredoxin pathway as summarized in Figure 1. The results demonstrate that not only expression of *TXNIP *and *TXNRD1*, the two factors influencing the thioredoxin pathway, but also downstream effectors show prognostic relevance in breast cancer (Table 4): thioredoxin (*TXN*), the M2 subunit of ribonucleotide reductase (*RRM2*), peroxiredoxin 2 (*PRDX2*), *HIF-1α *and *VEGF *were significantly associated with worse prognosis in the combined cohort as well as in at least one of the studied cohorts. The most convincing association was shown by *RRM2*, with similar results in all three individual cohorts. Ribonucleotide reductase depends on activation by thioredoxin in order to reduce ribonucleotides to deoxyribonucleotides, and therefore supports DNA synthesis.

**Table 4:** Association of other members and effectors of the thioredoxin pathway with metastasis-free survival

	Prognostic factor	*P *value	HR	95% CI
Mainz cohort (*n *= 200)	*TXN *RNA expression	0.001	3.029	1.599 to 5.738
	*RRM2 *RNA expression	0.001	1.598	1.205 to 2.120
	*PRDX2 *RNA expression	0.066	1.950	0.957 to 3.975
	*HIF1A *RNA expression	0.568	0.870	0.540 to 1.403
	*VEGFA *RNA expression	0.485	1.206	0.713 to 2.042
	*PTEN *RNA expression	0.313	0.774	0.470 to 1.273
Rotterdam cohort (*n *= 286)	*TXN *RNA expression	0.067	1.428	0.976 to 2.091
	*RRM2 *RNA expression	< 0.001	1.440	1.181 to 1.756
	*PRDX2 *RNA expression	0.234	0.767	0.496 to 1.187
	*HIF1A *RNA expression	0.003	1.545	1.160 to 2.058
	*VEGFA *RNA expression	0.907	0.982	0.724 to 1.332
	*PTEN *RNA expression	0.339	0.851	0.612 to 1.184
Transbig cohort (*n *= 302)	*TXN *RNA expression	0.069	1.411	0.974 to 2.043
	*RRM2 *RNA expression	0.003	1.354	1.107 to 1.655
	*PRDX2 *RNA expression	0.687	1.129	0.627 to 2.033
	*HIF1A *RNA expression	0.024	1.464	1.052 to 2.038
	*VEGFA *RNA expression	0.045	1.393	1.007 to 1.926
	*PTEN *RNA expression	0.197	0.802	0.573 to 1.121
Combined cohort (*n *= 788)	*TXN *RNA expression	< 0.001	1.755	1.436 to 2.145
	*RRM2 *RNA expression	< 0.001	1.469	1.298 to 1.663
	*PRDX2 *RNA expression	0.004	1.423	1.120 to 1.807
	*HIF1A *RNA expression	< 0.001	1.440	1.184 to 1.750
	*VEGFA *RNA expression	0.033	1.239	1.018 to 1.508
	*PTEN *RNA expression	0.243	0.885	0.720 to 1.087

Association of *TXN*, *RRM2*, *PRDX2*, *HIF1A*, *VEGF *and *PTEN *RNA expression with metastasis-free survival in patients with node-negative breast cancer in the univariate Cox analysis. In the Mainz cohort, *TXN *and *RRM2 *RNA expressions are associated with worse metastasis-free survival. In the Rotterdam cohort, *RRM2 *and *HIF1A *are associated with metastasis-free survival. In the Transbig cohort, significant associations were found for *RRM2*, *HIF1A *and *VEGFA*. In the combined cohort of 788 patients, a significant association of all factors with metastasis-free survival, except *PTEN*, was obtained. HR, hazard ratio; CI, confidence interval.

### TXNRD1 and TXNIP depend on ERBB2 expression in MCF-7 cells

**Figure 5:**
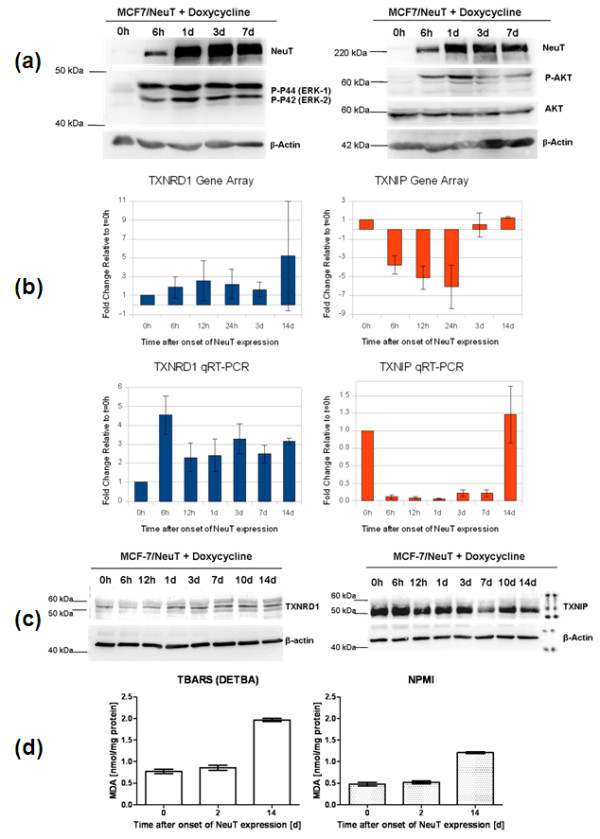
**Induction of ERBB2 (NeuT) by the Tet-on system in MCF-7/NeuT cells influences TXNRD1 and TXNIP**. **(a) **Oncogenic ERBB2 (NeuT) overexpression in MCF-7 cells, induced by exposure to doxycycline, triggers phosphorylation of ERK1/2 and AKT (PKB) as shown by immunoblotting. **(b) **ERBB2-mediated changes in the transcription of thioredoxin reductase 1 (*TXNDR1*) and thioredoxin interacting protein (*TXNIP*) are demonstrated both by Affymetrix Gene Array data and quantitative real-time PCR (qRT-PCR). The gene array graphs display the mean values (± standard deviation) of the Affymetrix probesets. The qRT-PCR graphs show a representative experiment of three with the mean value (± standard deviation) of a triplicate amplification. **(c) **TXNRD1 and TXNIP protein levels were analyzed by inmmunoblotting. Bands of ~55 kDa for TXNRD1 and ~50 kDa for TXNIP were observed. **(d) **An increase in accumulation of reactive oxygen species 14 days after onset of ERBB2 (NeuT) expression was shown by detection of malondialdehyde by a thiobarbituric acid reactive substance (TBARS) assay and an *N*-methyl-2-phenylindole (NMPI) assay. The results represent the mean (± standard deviation) of three experiments, each measured eight times. DETBA, 1,3-diethyl-2-thiobarbituric acid.

In order to analyze whether ERBB2 influences the levels of *TXNRD1 *and *TXNIP *expression, we used an MCF-7 cell line that allows doxycycline-dependent expression of an oncogenic version of ERBB2 (NeuT) by the Tet-on system [3,4]. Addition of doxycycline to the culture medium resulted in overexpression of oncogenic ERBB2 to levels seen in overexpressing breast carcinomas (Figure 5a). Induced ERBB2 overexpression was accompanied by increased phosphorylation of ERK1/2 and AKT/PKB, illustrating that doxycycline-induced ERBB2 is functional (Figure 5a).

As previously observed [3,4], overexpression of oncogenic ERBB2 also induced cell cycle arrest and premature senescence in MCF-7 cells, which is accompanied by several phenotypical changes as can be observed in Additional file 6. ERBB2-triggered premature senescence has been found to be mediated by the P21 protein [3], and *P21 *mRNA levels were increased as early as 6 hours after onset of NeuT expression (Additional file 7).

Interestingly, induction of ERBB2 influenced the expression of TXNIP and TXNRD1 in a way that was reproducible in three independent experiments: the *TXNIP *transcript was immediately downregulated during the first 24 hours after addition of doxycycline, but recovered to initial levels at later time intervals (Figure 5b); the *TXNRD1 *transcript was immediately upregulated after doxycycline addition and fluctuated during the observed period, with levels remaining clearly higher than the initial (Figure 5b); and the TXNIP and TXNRD1 proteins showed the same trend as the corresponding transcripts, as assayed by immunoblotting. The onset of the upregulation and downregulation appeared somewhat delayed compared with that of the transcripts (Figure 5c). In addition to this - as evidenced by analysis of the reaction of MDA with DETBA (thiobarbituric acid reactive substance assay) and with *N*-methyl-phenylindole (Figure 5d), as well as by a lipid peroxidation assay (Additional file 8) - ROS were not increased at day 2 but were clearly increased at day 14 after onset of NeuT expression.

In conclusion, induced overexpression of ERBB2 caused alterations of *TXNRD1 *and *TXNIP *expression, whereby *TXNRD1 *was upregulated and *TXNIP *downregulated. Both redox factors were influenced by ERBB2 in a way that, in our patient cohorts, was associated with worse prognosis.

## Discussion

Targeting thioredoxin reductase and thioredoxin has been suggested recently as a basis for cancer therapy, including breast cancer [22,39,40]. The prognostic role of TXNRD1 and TXNIP, key players of the thioredoxin system, in node-negative breast cancer, however, has not yet been analyzed. We therefore studied three cohorts of patients who have not been treated by chemotherapy. In order to understand the role of TXNRD1 and TXNIP for the natural history of breast cancer it seemed relevant to focus on untreated patients, because the thioredoxin system has been reported to influence sensitivity of tumor cells to chemotherapy [23,24,41]. We observed that high expression of *TXNRD1 *was associated with worse prognosis, whereas high *TXNIP *expression was associated with better prognosis. These results obtained in our cohort, the Mainz cohort [31], could be confirmed in two independent, previously published cohorts - the Rotterdam cohort [32] and the Transbig cohort [33,34].

The inverse association of *TXNRD1 *and *TXNIP *with MFI found in this study supports the hypothesis that the maintenance of an active thioredoxin system is advantageous to the tumor cells because it limits oxidative damage and enables them to survive. Conversely, the important ROS-independent roles of TXNIP and thioredoxin might as well account for the promotion of tumorigenesis. It is noteworthy that both factors have opposite effects on HIF-1α, in a way that thioredoxin favors its increase and TXNIP its destabilization [42,43]. Additional associations with MFI found for thioredoxin and downstream factors of the thioredoxin pathway, including *PRDX2*, *RRM2*, *HIF1A *and *VEGF*, confirm the importance of the thioredoxin system in breast cancer regardless of its ROS-related or unrelated roles.

To better understand the interrelation of TXNRD1 and TXNIP with biologically relevant processes in breast cancer, we compared their expression levels with previously published metagenes [31,35,36,38]. The most striking correlations were observed with the proliferation metagene, showing that *TXNDR1 *is expressed strongly in fast proliferating tumors whereas *TXNIP *is expressed at low levels, which is in accordance with published data [44-47]. This constellation fits to the negative prognostic influence of TXNRD1 and the favorable effect of TXNIP. In addition, the estrogen receptor metagene was inversely correlated with *TXNRD1*. *TXNRD1 *also correlated with the T-cell metagene, which was surprising because the latter was associated with better prognosis in our previous studies [31]. Thioredoxin is known to be secreted by leukocytes and may exhibit cytokine-like properties in the extracellular environment [48]. A long controversial debate has been whether immune cell infiltration leads to a better prognosis of breast cancer by an attack on the tumor cells or whether secreted cytokines may cause on adverse effect creating a microenvironment that favors tumor cell proliferation [31].

Multivariate analysis adjusted for the conventional clinical parameters serves to identify whether a new factor adds some independent prognostic information to the already established parameters. In the Transbig cohort, *TXNIP *was independent of the established clinical parameters, whereas *TXNRD1 *was not. Of course a lack of influence in the multivariate Cox model does not exclude biological relevance. If several genes are responsible for progression of tumors from pT1 to pT4 it is likely that tumor stage, and not one of the many genes influencing tumor stage, will be influential in the multivariate regression model. In the case of *TXNRD1*, associations were observed with tumor grade and pT stage, hormone receptor status and ERBB2 status. We found the latter association particularly interesting, because it might be explained by an influence of overexpressed ERBB2 on *TXNRD1 *expression levels. When we studied *TXNRD1 *expression in ERBB2-status-positive versus ERBB2-status-negative carcinomas, significantly higher levels were obtained for ERBB2-status-positive tumors in all three individual study cohorts. Conversely, the difference in *TXNRD1 *expression levels was relatively small. This observation is not surprising, however, since an influence of ERBB2 will probably represent only one of several factors influencing *TXNRD1 *expression.

Inducible expression systems with erbB2 in mice or cell lines have contributed a lot to our understanding of erbB2-associated mechanisms [49,50]. To understand whether ERBB2 causes the increase in *TXNRD1 *expression or whether their association represents only an epiphenomenon of other primary events, we therefore applied the MCF-7/NeuT cell line, which allows inducible expression of an oncogenic version of ERBB2 (NeuT) [3,4]. Interestingly, switching on ERBB2 caused a clear increase in TXNRD1 mRNA and protein levels. A link between ERBB2 and *TXNRD1 *expression has been established in rat cardiomyocytes [51] where neuregulin 1β, a ligand of the ERBB receptor tyrosine kinase, upregulated the expression of thioredoxin and TXNRD1, among that of other genes involved in protection against oxidative stress, both at the mRNA and protein levels. This link supports the possibility that the immediate early increase in *TXNRD1 *mRNA levels observed after NeuT overexpression in our MCF-7/NeuT cell system is a direct response to ERBB2 signaling. The high *TXNRD1 *levels observed at relatively long periods (14 days) after NeuT induction could be due to the accumulation of ROS at this later stage. Since it is known that 4-hydroxynonenal, one of the major products of lipid peroxidation, can increase *TXNRD1 *mRNA levels via transcriptional activation of the NF-E2-related factor 2 signaling pathway and that TXNRD1 promoter contains an antioxidant response element [52], it is plausible that the observed accumulation of ROS in our cells at day 14 is contributing to the higher *TXNRD1 *expression at this time point.

Surprisingly, TXNIP was also influenced by NeuT but followed a completely different kinetic, with an initial downregulation of *TXNIP *transcript levels within 24 hours and a later recovery at 14 days. The immediate decrease of *TXNIP *mRNA upon ERBB2 expression suggests again a direct link between both factors. To our knowledge, nothing has so far been reported concerning a direct influence of ERBB2 signaling on *TXNIP *mRNA expression. The later increase of *TXNIP *mRNA levels begins concomitant with the G_1 _arrest of the MCF-7 cells as they undergo senescence [3]. Although the molecular mechanisms leading to the *TXNIP *biphasic behavior remain unknown, it fits the inverse correlation of *TXNIP *with the proliferation metagene observed in our study and it agrees with the known function of the TXNIP gene as a tumor suppressor [42].

**Figure 6:**
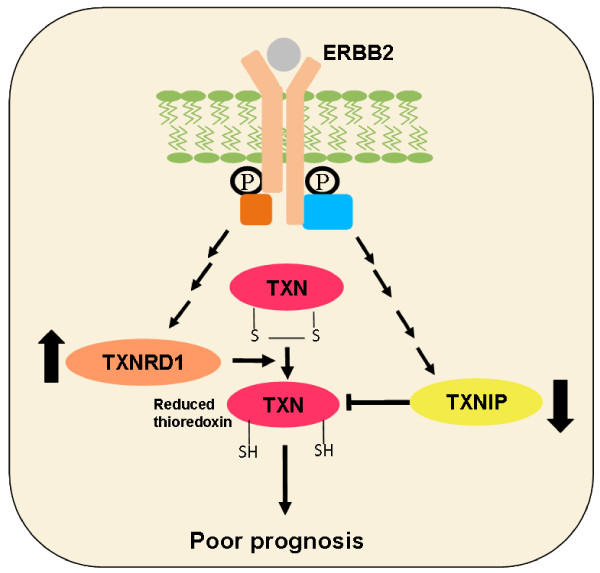
**ERBB2-mediated altered expression of *TXNRD1 *and *TXNIP *and the association with prognosis**. ERBB2 mediates upregulation of thioredoxin reductase 1 (*TXNDR1*) expression and downregulation of thioredoxin interacting protein (*TXNIP*) expression, which in turn are associated with bad prognosis in breast cancer. TXN, thioredoxin.

The observed initial activation of *TXNRD1 *and repression of *TXNIP *transcription by ERBB2 signaling independent of ROS in our MCF-7/NeuT cell model could be interpreted as part of an ERBB2-triggered survival program that intends to prevent later accumulation of ROS. Further, it suggests that TXNIP and TXNRD1 are ERBB2 effectors whose multiple cellular functions contribute to proliferation, apoptosis resistance, metabolic reprogramming and, finally, to the hallmarks of ERBB2-positive breast tumors (Figure 6).

Accumulation of ROS in senescent MCF-7/NeuT cells (14 days) is accompanied by the recovery of *TXNIP *mRNA to initial levels, which is expected to negatively influence the ROS-scavenging activity of thioredoxin. This could therefore partially explain increased ROS at this stage. Interestingly, mRNA expression of thioredoxin itself decreases slightly in MCF-7/NeuT cells at 14 days (Additional file 9). Many other factors, however, are also likely to contribute to the late accumulation of ROS in senescent cells. This is under current investigation.

## Conclusions

In the present article we have shown that high *TXNRD1 *expression and low *TXNIP *expression are associated with worse prognosis in node-negative breast cancer. ERBB2 can influence expression levels of *TXNRD1 *and *TXNIP *to shift balances in a prognostically unfavorable manner.

## Abbreviations

DETBA: 1,3-diethyl-2-thiobarbituric acid; GEO: Gene Expression Omnibus; HIF-1: hypoxia inducible factor 1; HR: hazard ratio; MDA: malondialdehyde; MFI: metastasis-free interval; NF: nuclear factor; PCR: polymerase chain reaction; ROS: reactive oxygen species; TGT: mean target intensity; TNXRD1: thioredoxin reductase 1; TXNIP: thioredoxin interacting protein; VEGF: vascular endothelial growth factor.

## Competing interests

MG is employed by Siemens Healthcare Diagnostics Products GmbH, which is in the business of commercializing diagnostic products. The other authors declare that they have no competing interests.

## Authors' contributions

CC and JGH contributed to the study conception and design, analysis and interpretation of collected data, and drafted and revised the manuscript critically for important intellectual content. DF, MSchu and LJM carried out the real-time PCR experiments in MCF-7 cells. MSchm collected patient-related tissue and data, and contributed to the design of the study. MG carried out the gene array expression analysis of patient tissue and contributed to the design of the study. MH carried out the gene array expression analysis of MCF-7/NeuT cells. BG and WS characterized the MCF-7/NeuT cell system and performed the immunoblot assays. ASc and CW carried out the lipid peroxidation assays. EF, KI and JR performed the statistical analysis. JIB and ASi participated in study design and coordination, and revised the manuscript for important intellectual content. All authors read and approved the final manuscript.

## Supplementary Material

Additional file 1 **Clinicopathological characteristics of node-negative breast cancer patients**. A pdf file containing a table that displays the clinicopathological characteristics of node-negative breast cancer patients from the Mainz, the Rotterdam and the Transbig cohorts. Click here for file

Additional file 2 **Frequency distribution of *TXNRD1 *and *TXNIP *RNA expression in the combined cohort**. A pdf file showing frequency distribution of *TXNRD1 *and *TXNIP *RNA expression in the combined cohort of 788 patients with node-negative breast cancer. Click here for file

Additional file 3 **Frequency distributions of estrogen receptor, ERBB2 and progesterone receptor RNA expression in the combined cohort**. A pdf file showing the frequency distributions of estrogen receptor, ERBB2 and progesterone receptor RNA expression in the combined cohort, allowing the identification of the cut-off points for dichotomization. Click here for file

Additional file 4 **Frequency distributions of estrogen receptor, ERBB2 and progesterone receptor RNA in the three individual cohorts**. A pdf file showing the frequency distributions of estrogen receptor, ERBB2 and progesterone receptor RNA in the three individual cohorts (Mainz, Rotterdam and Transbig). Click here for file

Additional file 5 **Association of *TXNRD1 *and *TXNIP *RNA expression with the established clinical parameters in the combined cohort**. A pdf file showing the association of *TXNRD1 *and *TXNIP *RNA expression with the established clinical parameters age, pT stage, grading, hormone receptor as well as ERBB2 status in the combined cohort of 788 patients with node-negative breast cancer. Click here for file

Additional file 6 **Phenotypical alterations of MCF-/NeuT cells in the first 3 days after NeuT induction**. A videoclip showing phenotypical alterations of MCF-/NeuT cells in the first 3 days after NeuT induction. The transition from the proliferating into the senescent state is evident. Click here for file

Additional file 7 **Upregulation of *P21 *in doxycycline-induced, ERBB2 (NeuT)-overexpressing MCF-7 cells**. A pdf file showing upregulation of *P21 *in doxycycline-induced, ERBB2 (NeuT)-overexpressing MCF-7 cells as assayed by quantitative real-time PCR. Click here for file

Additional file 8 **Accumulation of ROS 14 days after onset of ERBB2 (NeuT) overexpression in doxycycline-induced MCF-7 cells**. A pdf file showing increase in the accumulation of ROS 14 days after onset of ERBB2 (NeuT) overexpression in doxycycline-induced MCF-7 cells, as determined by the lipid peroxidation assay LPO-586 (Oxis Research). Click here for file

Additional file 9 **RNA levels of thioredoxin**. A pdf file showing RNA levels of thioredoxin, assayed by Affymetrix gene arrays in MCF-7/NeuT cells. Click here for file
