# Supplementary material for: Role of thioredoxin reductase 1 and thioredoxin interacting protein in prognosis of breast cancer
Source: Breast Cancer Res. 2010 Jun 28;12(3):R44. doi: 10.1186/bcr2599 (PMC2917039; doi:10.1186/bcr2599)
Supplement: Additional file 1 — Clinicopathological characteristics of node-negative breast cancer patients. A pdf file containing a table that displays the clinicopathological characteristics of node-negative breast cancer patients from the Mainz, the Rotterdam and the Transbig cohorts. [file bcr2599-S1.PDF]

Additional file 1

Supplemental Table 1a. Clinicopathological characteristics of node negative breast cancer patients from the Mainz cohort (n=200).

Characteristics	n	%
**Age at diagnosis**		
<50	49	24.5
≥50	151	75.5
**pT stage**		
pT_1	112	56
pT_2	85	42.5
pT_3	3	1.5
**Histological grade**		
G I	42	21
G II	109	54.5
G III	48	24
**Estrogen receptor status**		
Negative	32	16
Positive	168	84
**Progesterone receptor status**		
Negative	68	34
Positive	132	66
**Hormone receptor status**[1]		
Negative	28	14
Positive	172	86
**HER-2 status**		
Negative	180	90
Positive	20	10
**Death**		
Of cancer	31	16.0
Unrelated to cancer	25	12.9
Surviving	143	71.5
**Relapse**	58	29
Regional	21	10.8
Metastasis	47	23.5
Contralateral	5	2.6
No relapse	136	70.1

[1]The hormone receptor status is positive as soon as one of both, the estrogen or the progesterone receptor status, is positive.

Supplemental Table 1b. Clinicopathological characteristics of node negative breast cancer patients from the Rotterdam cohort (30) (n=286). Estrogen receptor, progesterone receptor and HER-2 status were derived from the gene array data. Cutpoints were 10 for the estrogen receptor, 12.6 for HER-2 (ERBB2) and 4.9 for the progesterone receptor.

Characteristics	n	%
**Estrogen receptor**		
RNA expression low	79	27.6
RNA expression high	207	72.4
**Progesterone receptor**		
RNA expression low	136	47.6
RNA expression high	150	52.4
**Hormone receptor status**[1]		
RNA expression low	61	21.3
RNA expression high	225	78.7
**HER-2 status**		
RNA expression low	234	81.8
RNA expression high	52	18.2
**Metastasis**		
Yes	107	37.4
No	179	62.6

[1]The hormone receptor status is positive as soon as one of both, the estrogen or progesterone receptor RNA expression is high.

Supplemental Table 1c. Clinicopathological characteristics of node negative breast cancer patients from the Transbig cohort (31, 32) (n=302). Estrogen receptor, progesterone receptor and ERBB2 status were derived from the gene array data. Cutpoints were 10 for the estrogen receptor, 12.6 for HER-2 and 4.9 for the progesterone receptor.

Characteristics	n	%
**Age at diagnosis**		
<50	174	57.6
≥50	128	42.2
**pT stage**		
pT_1	163	54.0
pT_2	138	45.7
pT_3	1	0.3
**Histological grade**		
G I	60	19.9
G II	117	38.7
G III	106	35.1
not documented	19	6.3
**Estrogen receptor**		
RNA expression low	88	29.1
RNA expression high	214	70.9
**Progesterone receptor**		
RNA expression low	128	42.4
RNA expression high	174	57.6
**Hormone receptor status**[1]		
Negative	75	24.8
Positive	227	75.2
**HER-2 status**		
RNA expression low	262	86.8
RNA expression high	40	13.2
**Metastasis**		
Yes	78	25.8
No	224	74.2

[1]The hormone receptor status is positive as soon as one of both, the estrogen or progesterone receptor RNA expression is high.
