# Supplementary material for: Role of thioredoxin reductase 1 and thioredoxin interacting protein in prognosis of breast cancer
Source: Breast Cancer Res. 2010 Jun 28;12(3):R44. doi: 10.1186/bcr2599 (PMC2917039; doi:10.1186/bcr2599)
Supplement: Additional file 2 — Frequency distribution of TXNRD1 and TXNIP RNA expression in the combined cohort. A pdf file showing frequency distribution of TXNRD1 and TXNIP RNA expression in the combined cohort of 788 patients with node-negative breast cancer. [file bcr2599-S2.PDF]

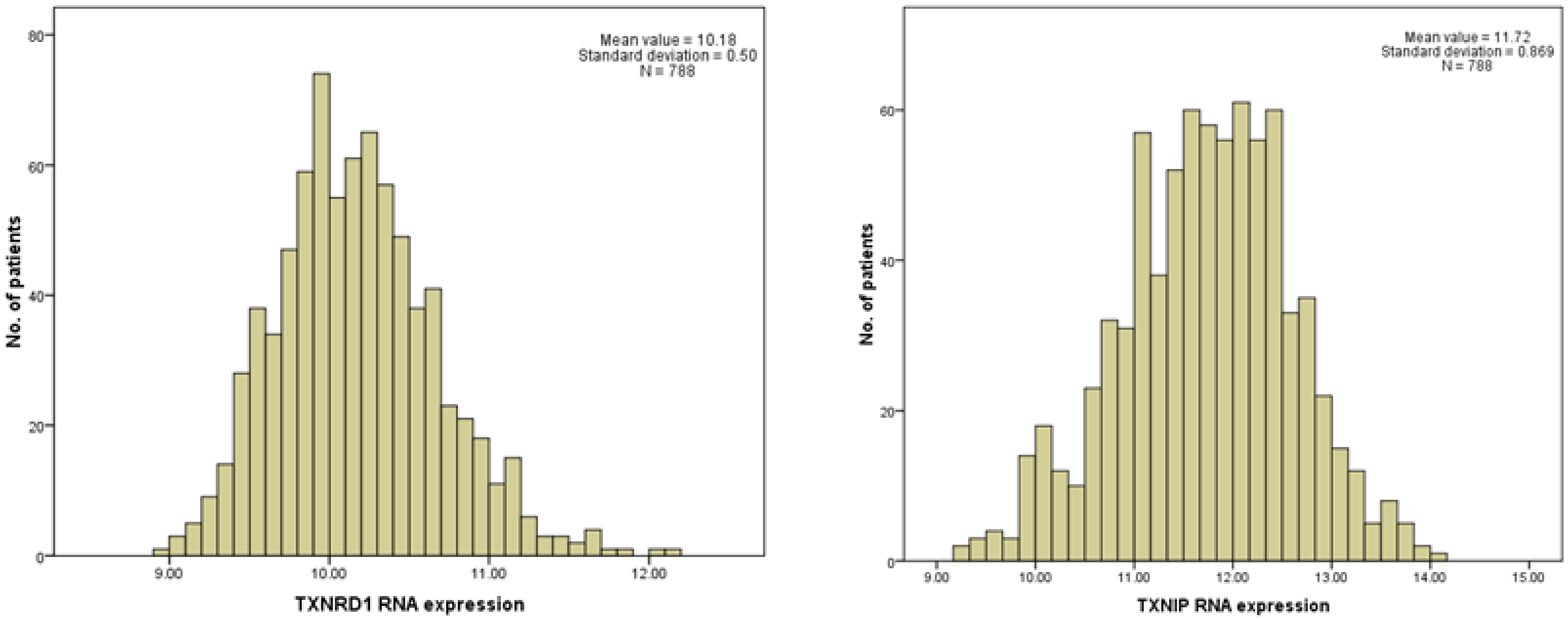

Additional file 2: Frequency distribution of TXNRD1 and TXNIP RNA expression in 788 patients with node-negative breast cancer. Since the frequency distributions did not perfectly match a normal distribution we used non-parametric tests for the further statistical analysis, such as the Mann-Whitney test for unpaired data.
