# Supplementary material for: Role of thioredoxin reductase 1 and thioredoxin interacting protein in prognosis of breast cancer
Source: Breast Cancer Res. 2010 Jun 28;12(3):R44. doi: 10.1186/bcr2599 (PMC2917039; doi:10.1186/bcr2599)
Supplement: Additional file 3 — Frequency distributions of estrogen receptor, ERBB2 and progesterone receptor RNA expression in the combined cohort. A pdf file showing the frequency distributions of estrogen receptor, ERBB2 and progesterone receptor RNA expression in the combined cohort, allowing the identification of the cut-off points for dichotomization. [file bcr2599-S3.PDF]

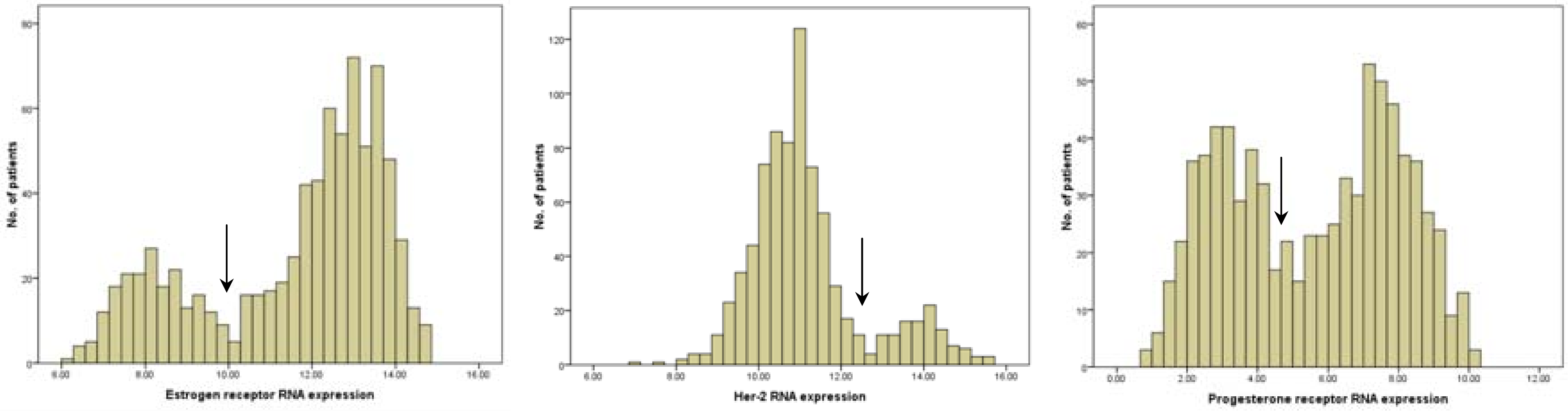

Additional file 3: Frequency distributions of estrogen receptor, ERBB2 (Her-2) and progesterone receptor RNA expression in the combined cohort. All three factors showed a bimodal distribution. The arrows indicate the cutpoints for dichotomization.
