# Supplementary material for: Role of thioredoxin reductase 1 and thioredoxin interacting protein in prognosis of breast cancer
Source: Breast Cancer Res. 2010 Jun 28;12(3):R44. doi: 10.1186/bcr2599 (PMC2917039; doi:10.1186/bcr2599)
Supplement: Additional file 4 — Frequency distributions of estrogen receptor, ERBB2 and progesterone receptor RNA in the three individual cohorts. A pdf file showing the frequency distributions of estrogen receptor, ERBB2 and progesterone receptor RNA in the three individual cohorts (Mainz, Rotterdam and Transbig). [file bcr2599-S4.PDF]

A. Mainz cohort (n=200)

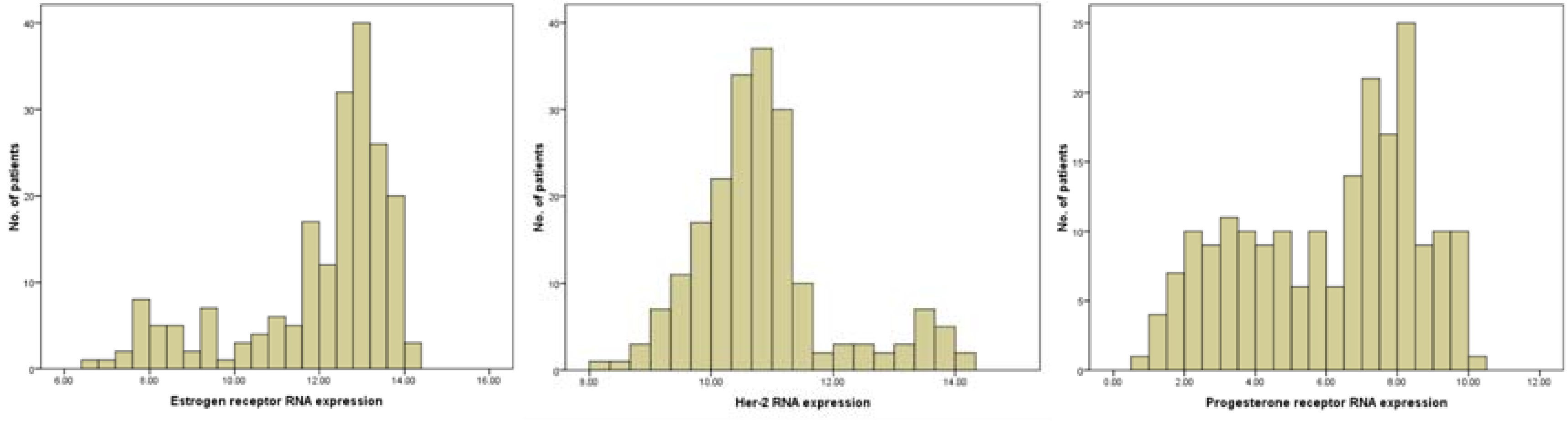

Additional file 4: Frequency distributions of estrogen receptor, ERBB2 (Her-2) and progesterone receptor RNA expression in the three individual cohorts. A. Mainz, B. Rotterdam, C. Transbig cohorts.

B. Rotterdam cohort (n=286)

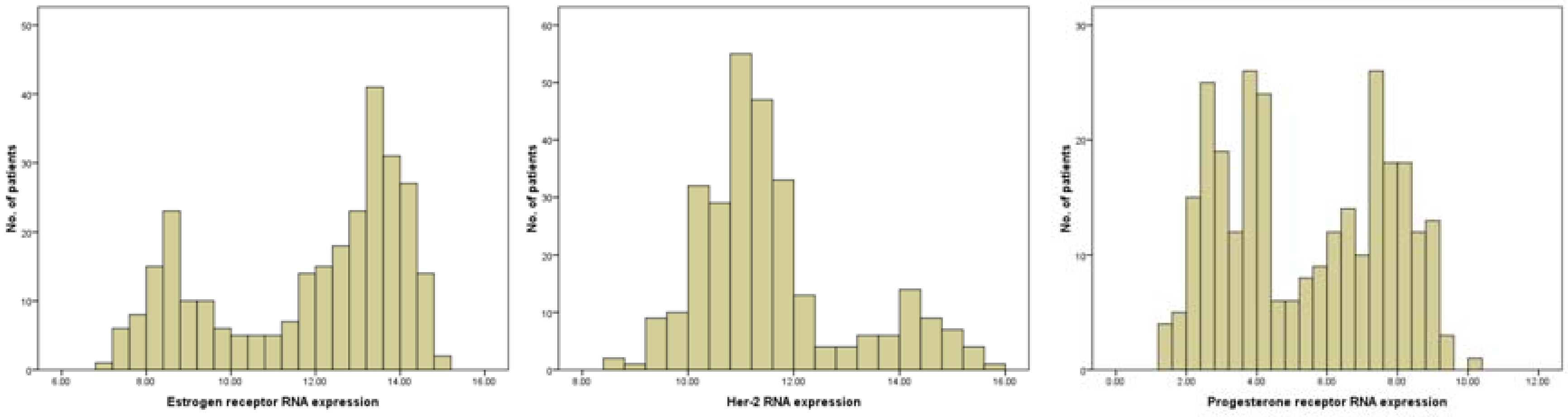

Additional file 4: continued

C. Transbig cohort (n=302)

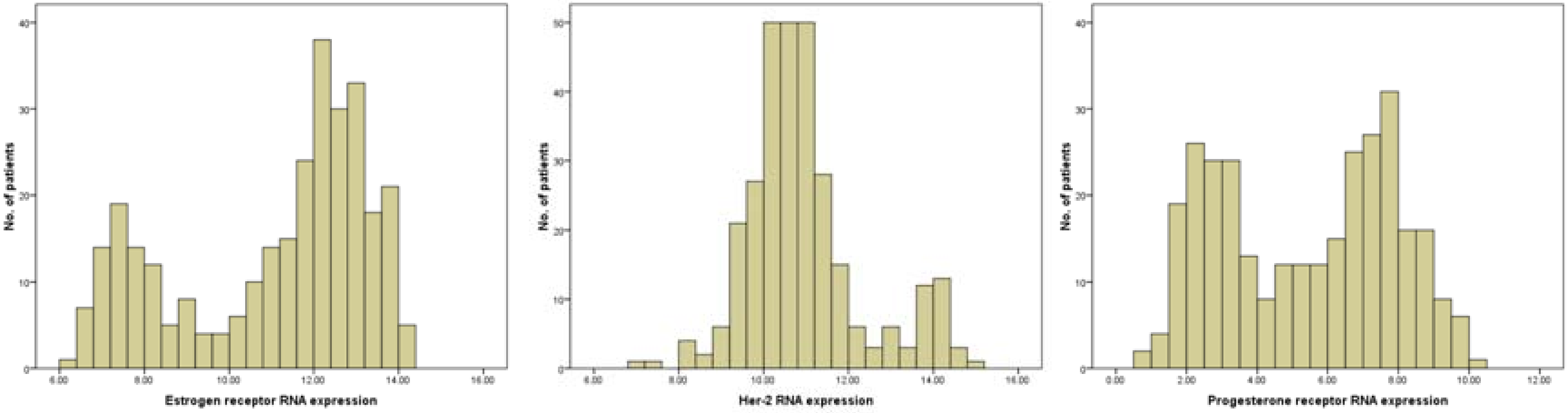

Additional file 4: continued
