# Supplementary material for: Role of thioredoxin reductase 1 and thioredoxin interacting protein in prognosis of breast cancer
Source: Breast Cancer Res. 2010 Jun 28;12(3):R44. doi: 10.1186/bcr2599 (PMC2917039; doi:10.1186/bcr2599)
Supplement: Additional file 5 — Association of TXNRD1 and TXNIP RNA expression with the established clinical parameters in the combined cohort. A pdf file showing the association of TXNRD1 and TXNIP RNA expression with the established clinical parameters age, pT stage, grading, hormone receptor as well as ERBB2 status in the combined cohort of 788 patients with node-negative breast cancer. [file bcr2599-S5.PDF]

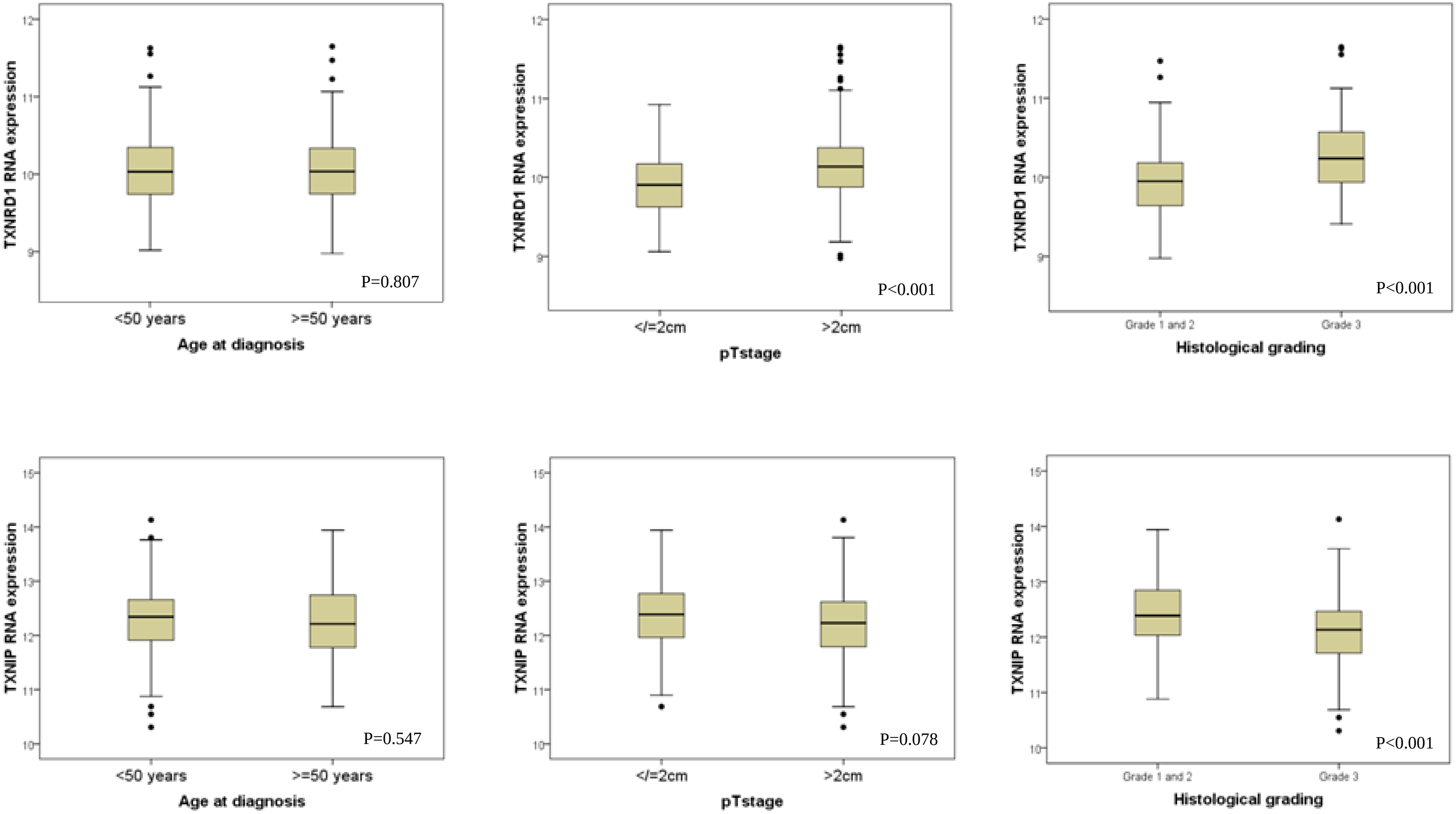

Additional file 5: Association of TXNRD1 and TXNIP RNA expression with the established clinical parameters age, pT stage, grading, hormone receptor as well as ERBB2 status in 788 patients with node-negative breast cancer. TXNRD1 was significantly associated with pT stage, grading, hormone receptor and ERBB2 status. TXNIP was significantly associated with grading and hormone receptor status. The Mann-Whitney test for unpaired data was applied to evaluate differences between the groups.

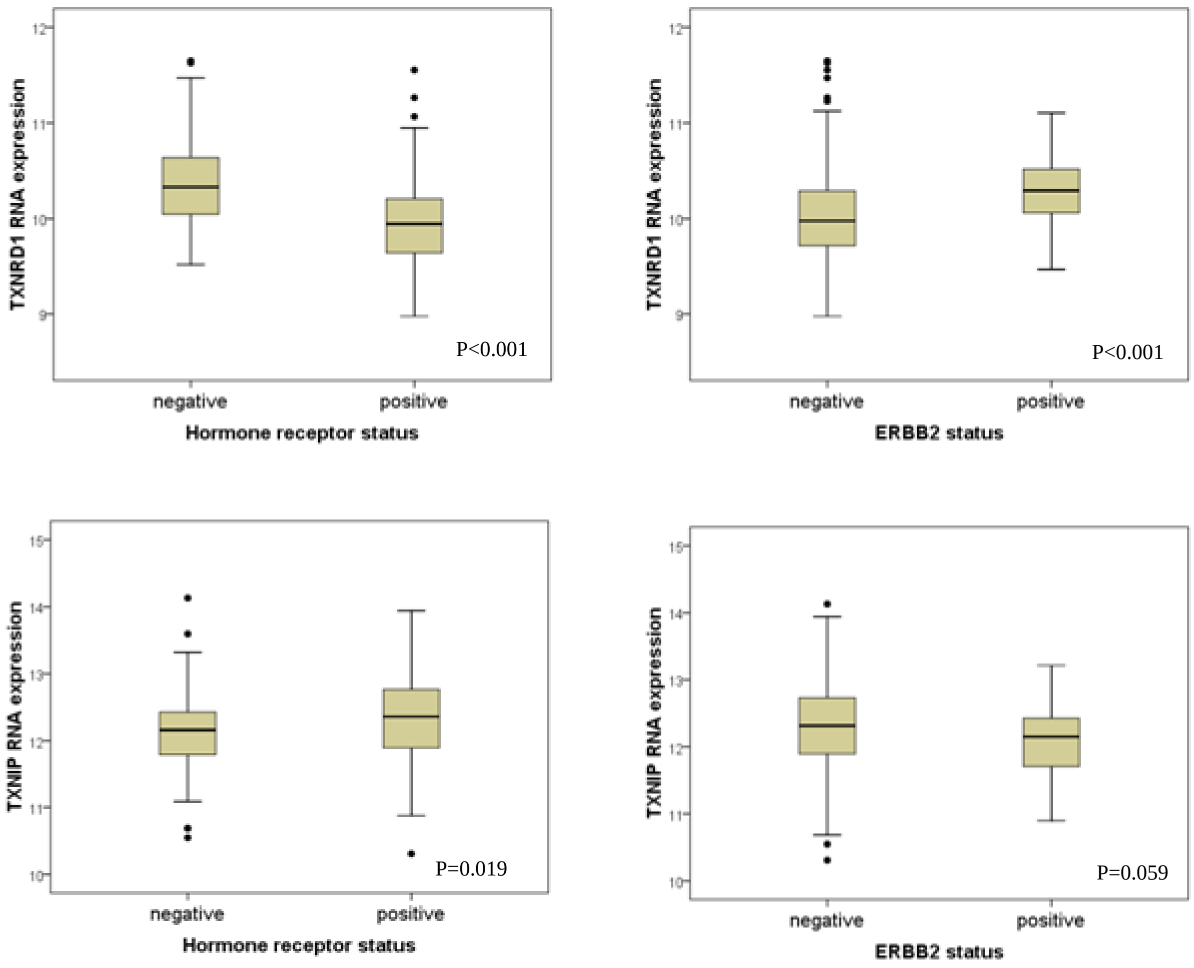

Additional file 5: continued
