# Supplementary material for: Role of thioredoxin reductase 1 and thioredoxin interacting protein in prognosis of breast cancer
Source: Breast Cancer Res. 2010 Jun 28;12(3):R44. doi: 10.1186/bcr2599 (PMC2917039; doi:10.1186/bcr2599)
Supplement: Additional file 7 — Upregulation of P21 in doxycycline-induced, ERBB2 (NeuT)-overexpressing MCF-7 cells. A pdf file showing upregulation of P21 in doxycycline-induced, ERBB2 (NeuT)-overexpressing MCF-7 cells as assayed by quantitative real-time PCR. [file bcr2599-S7.PDF]

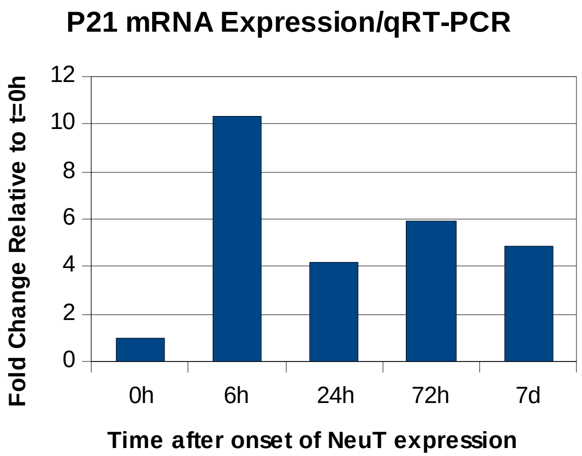

Additional file 7: Oncogenic ERBB2 (NeuT) overexpression in MCF-7 cells, induced by exposure to doxycycline, triggers upregulation of P21 which is involved in the process of oncogene-mediated premature senescence [3,4].
