# Supplementary material for: Role of thioredoxin reductase 1 and thioredoxin interacting protein in prognosis of breast cancer
Source: Breast Cancer Res. 2010 Jun 28;12(3):R44. doi: 10.1186/bcr2599 (PMC2917039; doi:10.1186/bcr2599)
Supplement: Additional file 8 — Accumulation of ROS 14 days after onset of ERBB2 (NeuT) overexpression in doxycycline-induced MCF-7 cells. A pdf file showing increase in the accumulation of ROS 14 days after onset of ERBB2 (NeuT) overexpression in doxycycline-induced MCF-7 cells, as determined by the lipid peroxidation assay LPO-586 (Oxis Research). [file bcr2599-S8.PDF]

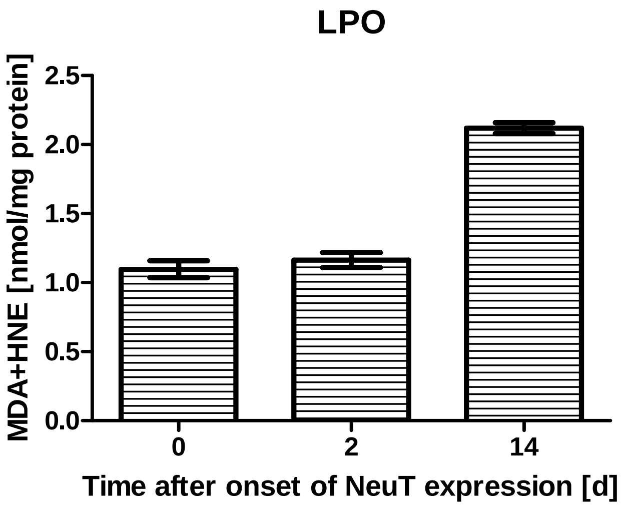

Additional file 8: The lipid peroxidation assay **(**LPO-586, Oxis research) shows an increase in accumulation of ROS 14 days after onset of ERBB2 (NeuT) expression in MCF-7 cells by measuring a combination of malonaldehyde (MDA) and 4-hydroxyalkenals.
