# Supplementary material for: Role of thioredoxin reductase 1 and thioredoxin interacting protein in prognosis of breast cancer
Source: Breast Cancer Res. 2010 Jun 28;12(3):R44. doi: 10.1186/bcr2599 (PMC2917039; doi:10.1186/bcr2599)
Supplement: Additional file 9 — RNA levels of thioredoxin. A pdf file showing RNA levels of thioredoxin, assayed by Affymetrix gene arrays in MCF-7/NeuT cells. [file bcr2599-S9.PDF]

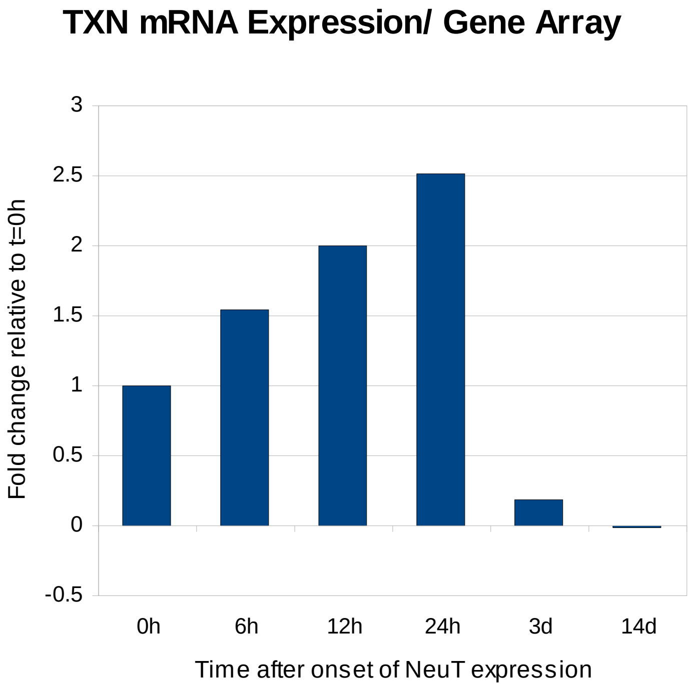

Additional file 9: Oncogenic ERBB2 (NeuT) overexpression in MCF-7 cells, induced by exposure to doxycycline, triggers changes in mRNA levels of thioredoxin, which is involved in ROS scavenging.
